# ZNF213 Facilitates ER Alpha Signaling in Breast Cancer Cells

**DOI:** 10.3389/fonc.2021.638751

**Published:** 2021-03-10

**Authors:** Huijie Yang, Xulei Lv, Xin Li, Lanzhi Mao, Zhiguo Niu, Ting Wang, Ting Zhuang, Qingsong Huang

**Affiliations:** ^1^ Department of Pharmacology and Tianjin Key Laboratory of Inflammation Biology, School of Basic Medical Sciences, Tianjin Medical University, Tianjin, China; ^2^ Xinxiang Key Laboratory of Tumor Migration, Invasion and Precision Medicine, Henan Key Laboratory of Immunology and Targeted Drugs, School of Laboratory Medicine, Henan Collaborative Innovation Center of Molecular Diagnosis and Laboratory Medicine, Xinxiang Medical University, Xinxiang, China; ^3^ Department of Anesthesiology, The Fourth Medical Center of Chinese PLA General Hospital, Beijing, China

**Keywords:** ZNF213, ER alpha, breast cancer, ubiquitin, stability

## Abstract

**Background:**

Breast cancer is the most common women malignancy worldwide, while estrogen receptor alpha positive type accounts for two third of all breast cancers. Although ER alpha positive breast cancer could be effectively controlled by endocrine therapy, more than half of the cases could develop endocrine resistance, making it an important clinical issue in breast cancer treatment. Thus, decoding the detailed mechanism, which controls ER alpha signaling activation and ER alpha protein stability, is of great importance for the improvement of breast cancer therapy. Several zinc finger proteins were shown to mediate the ubiquitination process and modulate protein stability. Thus, we further explore the function of Zinc finger protein 213 on ER alpha protein stability and tamoxifen resistance.

**Methods:**

CCK8 and Edu assay was used to measure cell proliferation. RNA sequence was performed by Ingenuity pathway analysis. The ER alpha signaling activities were measured with luciferase assay, real-time quantitative PCR, and western blotting. Protein stability assay and ubiquitin assay were used to determine ER alpha protein degradation and ubiquitination. The immuno-precipitation was utilized to determine ER alpha and ZNF213 interaction. The ubiquitin-based immuno-precipitation assay was sued to detect specific ubiquitination manner on ER alpha.

**Results:**

We identified ZNF213 as a novel zinc finger protein, which modulated ER alpha protein. ZNF213 expression correlated with poor outcome in endocrine treated patients. ZNF213 depletion inhibited ER alpha signaling and proliferation in breast cancer cells. Further mechanistic studies showed ZNF213 located in cytosol and nuclear, which modulated ER alpha stability *via* inhibiting ER alpha K48-linked ubiquitination.

**Conclusions:**

Our study reveals an interesting post-translational mechanism between ER alpha and ZNF213 in breast cancer. Targeting ZNF213 could be an appealing strategy for ER alpha positive breast cancer.

## Introduction

Breast cancer ranks NO.1 in women cancer incidence in the world (1). According to recent cancer statistics, approximately 1.6 million newly diagnosed breast cancer cases each year, which accounts for about 20% of all women cancer incidence (2). According to the clinical-pathological classification, breast cancer can be divided into three groups: endocrine receptor-positive (estrogen receptor or progesterone receptor), HER2-positive (human epidermal growth factor receptor 2), and triple negative breast cancer (ER, PR, and HER2) (3). A majority (~70%) of breast tumors are estrogen receptor positive, and a significant portion (~90%) of ER-positive (ER^+^) breast tumors are also androgen receptor-positive (AR^+^) which is found to be predominantly expressed in *in-situ*, invasive, and metastatic breast cancers (4). Compared with HER2 positive and triple negative breast cancer subtypes, ER alpha positive breast cancer patients show a significant priority in prognosis and could benefit from endocrine therapy (5). However, more than half of the patients develop endocrine resistance during the treatment, which becomes a major challenge in both the basic research and clinics (6). Thus, decoding the potential mechanism, which controls ER alpha expression coupled with ER alpha protein stability, is of great importance to characterize endocrine resistance mechanism.

Estrogen receptor alpha (ER) was firstly discovered in 1985, which belongs to the super family of nuclear receptors (7). ER alpha protein is composed of three functional domains: Activation Function 1 (AF1) domain, DNA-binding domain (DBD), and Activation function 2 (AF2) domain (8). The AF1 domain is responsible for the interaction with co-activators, while the AF2 domain mediates the association with ER alpha ligands. When ER alpha binds to its ligands, the ER alpha protein could trans-locate into the nuclear and form dimers, which subsequently binds to the promoter regions of ER alpha target genes (9). The aberrant activation of ER alpha signaling has been regarded as the driver pathway for most of ER alpha positive breast cancers (10). Besides, the activation through ER alpha ligands, the ER alpha signaling could also been modified through post-translational modifications. For example, P300 could induce the acetylation of ER alpha protein at the hinge domain and enhance ER alpha signaling function (11). Besides, SRC kinase could promote ER alpha phosphorylation at Y537 sites, which induce the conformation change of ER alpha and tamoxifen resistance (12). Recent studies demonstrated several RING finger proteins could also modulate ER alpha function and breast cancer proliferation (13, 14). One example is that SHARPIN/RNF31 modulates ER alpha protein stability and ER alpha signaling activity in breast cancer cells (15, 16). Based on the previous studies, we can propose that the ER alpha interaction proteins might control the ER alpha ubiquitination, protein stability, and also ER alpha signaling activity.

Zinc finger domains (ZNF) are small protein motifs, containing about 50 residues (17). The zinc finger domains modulate the protein-protein interactions (18). Although ZNF proteins were shown to directly bind DNA/RNA and modulated gene expression (19), several zinc finger proteins were shown to mediate the ubiquitination process and modulate protein stability (14, 16). Our current study identifies ZNF213 (Zinc finger protein 213) as an important modulator for ER alpha signaling. ZNF213 is elevated in breast cancers and relates to poor prognosis in endocrine treated patients. Besides, ZNF213 facilitates ER alpha signaling and breast cancer progression *via* enhancing ER alpha stability. Our finding provides a novel insight of ZNF family members in mediation cancer progression and nuclear receptor function. Targeting ZNF213 could be a promising strategy for breast cancer therapy.

## Materials and Methods

### Cell Culture

MCF-7, T47D, and HEK293 cells are got form American Type Culture Collection (ATCC). T47D cells are cultured with RPMI-1640 (42401, Life Technologies) supplemented with 2 mM L-glutamine (25030, Life Technologies) and 10% FBS. MCF-7 and HEK293 cells are cultured with Dulbecco’s Modified Eagle’s Medium that contains 4,5 g/L glucose and 4 mM L-glutamine (DMEM, 41965, Life Technologies) supplemented with 10% Fetal Bovine Serum (FBS, 10270, Life Technologies). All cell lines are cultured in 1% penicillin/streptomycin (Invitrogen). All cell lines are characterized by cell line authentication. The cell line authentication *via* Short Tandem Repeat (STR) is performed *via* PowerPlex 21 system. The STR data of MCF-7 and T47D cell lines are found consistent with STR data in ATCC.

### Plasmids and siRNA

The Myc-ZNF231 plasmid is acquired from Origene Company (https://www.origene.com). The ZNF213 deletion constructs were sub-cloned from the full-length plasmid. The ER alpha full and deletion constructs were described in previous study. The HA-K48 and HA-Ub plasmids were gifted from Dr. Bo Yang and Jie Wang (20). The Estrogen-Response-Element (ERE)-TK reporter and Renilla plasmids were used in previous study (21) and are transfected with Lipofectamin 2000 (1662298, Invitrogen). For siRNA transfection, the ZNF213 siRNA sequences are #1: 5’-GGAUCUCUUCUGGGACAUA-3’, 5’-UAUGUCCCAGAAGAGAUCC-3’;

#2: 5’-GGCAUUGGGAGACAUCCCA-3’, 5’-UGGGAUGUCUCCCAAUGCC-3’. The siControl sequences are 5’-UUCUCCGAACGUGUCACGUTT-3’, 5’-ACGUGACACGUUCGGAGAATT-3’.

### RNA Extraction and qPCR Analysis

RNeasy plus mini kits were used to extract total RNA (Qiagen) (22). The RNA concentration was measured *via* Nanodrop. The RNA quality was pre-checked *via* 18S/28S ratio in 1% agarose gel. Real-time PCR was performed as previously described (23). 36B4 was used as internal control. Primer sequences for qPCR are shown in **Table 1**.

**Table 1:** Primer sequence information for Q-PCR.

PS2 F 5‐cat cga cgt ccc tcc aga aga g‐3 PS2 R 5‐ctc tgg gac taa tca ccg tgc tg‐3
PDZK1 F 5‐gcc agg ctc att cat caa aga‐3 PDZK1 R 5‐cct cta gcc cag cca agt ca‐3
PKIB F 5‐ gag tct ggg gtc gcc aat‐3 PKIB F 5‐tga act ctg gat gtctgg taa gg‐3
GREB1 F 5‐cgt gtg gtg act gga gta gc‐3 GREB1 R 5‐acc tct tca aag cgt gtc gt‐3
ZNF213 F 5‐ gcg acc ctg gag tac aca tc‐3 ZNF213 R 5‐tca tgc tgg gca gat tcc tg‐3
36B4 F 5‐ggc gac ctg gaa gtc caa ct‐3 36B4 R 5‐cca tca gca cca cag cct tc‐3

### Quantification of Cell Viability

MCF-7 and T47D cells were transfected with siZNF213 or siControl in 24-well plate. Twenty-Four hours after transfection, the cells number was countered and 4,000 cells were seeded into 96-well plates. The relative cell viability was measured at indicated time points. Cell numbers were determined using the CCK8 cell proliferation reagent as previously described (24).

### EdU Assay

Cell proliferation was determined by EdU (5-ethynyl-20 -deoxyuridine) assay using EdU Cell Proliferation Assay Kit (Ribobio, Guangzhou, China). MCF-7 and T47D cells were seeded in 96-well plates for transfection with siControl and siZNF213. After 24 h, cells were added with 50 mM EdU and continued incubating for another 2 h. Then Using 4% paraformaldehyde to fix cells and Apollo Dye Solution to stain with proliferating cells. Nucleic acids were stained with Hoechst 33342. The cell proliferation rate was calculated according to the imageJ.

### Wound Healing Assay

MCF-7 and T47D cells were seeded and transfected with 30 nM ZNF213 siRNA or Control siRNA. Then 24 h after transfection, cells were seeded into six-well plates with 1% FBS. When the cells were 100% fused, we scratched the cells with the tips of yellow pipette. The wound distance was measured at indicated time points and normalized with starting time point. Percentage wound recovery was expressed as: [1-(Width of the wound at a given time/width of the wound at t = 0)] × 100%.

### Western Blotting

Cells were harvested and lysed with RIPA buffer with cOmplete™ protease Inhibitor cocktail (Roche 11873580001). Proteins were separated by electrophoresis on SDS-polyacrylamide gel electrophoresis (PAGE) and electro-transferred to PVDF membrane. The antibodies used in this study were listed here: Anti-ZNF213 (HAP035000, Sigma); Anti-ER alpha (D8H8, 8644, Cell signaling Technology); Anti-HA (MMS-101R, COVANCE); Anti-myc (9E10, ab32, Abcam); Anti-myc (Ab9106, Abcam); Anti-Actin (A5441, Sigma); Anti-Flag (20543-1-AP, Proteintech); Anti-GFP (Ab290, Abcam). Membranes were then washed with PBS for three times and incubated with secondary antibodies Peroxidase-Conjugated AffiniPure Goat Anti-Mouse IgG (Beyotime A0216) or Goat Anti-Rabbit IgG (Beyotime A0208). Fluorescent signals were visualized with ECL system (Amersham imager 600, USA).

### Luciferase Assay

The luciferase activity of estrogen signaling activity was performed using the Dual-Luciferase Reporter kit (Promega, Germany). The ERE luciferase reporter was transfected together with the Renilla plasmid into the cells. Luciferase activity was measured after 24 h.

### Co-Immunoprecipitation Assay

Immunoprecipitation was performed as described in previous study (25). The MCF-7 total cell lysis were pre-cleared with rabbit IgG for 2 h and subsequently immunoprecipitated with ER alpha antibody (SC8005, Santa Cruz) overnight, while rabbit IgG (Santa Cruz) was used as the negative control. The bounded protein was analyzed by Anti-ZNF213 (HAP035000, Sigma). For the overexpression experiment, HEK293 cells were transfected with 5ug GFP-ZNF213 (Full length or deletion domains) and ER alpha plasmid (Full length or deletion domains) in 10 cm dish. Cell lysates were pre-cleared with IgG and subsequently incubate with GFP (Ab290, Abcam) antibody, while rabbit IgG was used as the negative control. The bound proteins were analyzed by western blotting.

### Poly-Ubiquitination Detection Assay

To directly detect the enriched overall ubiquitinated or K48-ubiqutinated ER alpha from the cell extracts, HEK293 cells were transfected with 4 ug Ub or 4 ug K48 Ubi plasmid, 2 ug ER alpha together with 0.5 ug Myc-ZNF213 or Myc-vector. After 48 h, total protein was extracted and pre-cleared with 20 ul protein A (santacruz, SC-2001) for 2 h. The supernatant was collected and immunoprecipitated by ER alpha antibody. Western blot with HA antibody was performed to detect total and K48 poly-ubiquitinated ER alpha.

### Immunofluorescence Assay

MCF-7 cells were fixed with 4% paraformaldehyde in PBS for 10 min, permeabilized with 0.2% Triton X-100 for 5 min, and blocked by 5% BSA in PBS for 1 h. A rabbit anti-ZNF213 (HAP035000, Sigma) and mouse anti-ER alpha monoclonal antibodies (SC-56833) were used, followed by Alexa Flour 647 (Invitrogen) anti-rabbit antibody and FITC-conjugated anti-mouse antibodies (Jackson ImmunoResearch, West Grove, PA, USA). As negative controls, the samples were incubated with the secondary antibodies without primary antibodies. Images were acquired under conditions fulfilling the Nyquist criterion using Nikon A+ laser scanning confocal system with a 60× oil NA1.4 objective and pinhole size of 1.0 Airy Unit. The acquired pictures were further processed and assembled using Image J.

### RNA Sequence Analysis

The global gene expression analysis (siControl and siZNF213) was based on RNA sequencing platform from BGI (Beijing Genomic Institute). The RNA sequence data are deposited in the Gene Expression Omnibus (GEO) database (Assessing number: GSE143948). Analysis was performed for differentially expressed genes (P < 0.01 and fold change >2) by Ingenuity Pathway Analysis (IPA).

### Statistics

Student’s t-test, Pearson correlation coefficient, and Cox regression analysis were used for comparisons. A P-value of <0.05 was considered to be significant.

## Results

### ZNF213 Is Elevated in Human Breast Cancer and Relates to Poor Prognosis in Endocrine Therapy Patients

**Figure 1:**
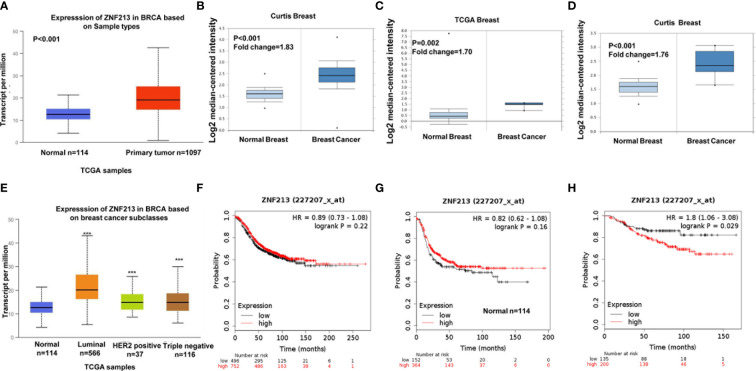
ZNF213 is elevated in human breast cancer and relates to poor prognosis in endocrine therapy patients **(A–D)** ZNF213 expression is elevated in breast cancer compared with normal breast tissues in multiple database (https://tcga-data.nci.nih.gov/docs/publications/tcga/) and (https://www.oncomine.org/). **(E)** Comparison of expression levels of ZNF213 in normal tissues and different types of breast cancer tissues in the TCGA database (https://tcga-data.nci.nih.gov/docs/publications/tcga/). ***P < 0.001. **(F)** ZNF213 has no prognostic impact on ER alpha positive breast cancer. **(G)** ZNF213 has no prognostic impact on ER alpha negative breast cancer. **(H)** ZNF213 expression relates to poor prognosis in endocrine therapy patients.

Firstly, we investigated the ZNF213 expression in public available database. From the TCGA database (https://tcga-data.nci.nih.gov/docs/publications/tcga/) and ONCOMINE database (https://www.oncomine.org), we found ZNF213 was significantly elevated in breast cancer tissues compared with normal breast tissues (**Figures 1A–D**). When we further analyzed the expression of ZNF213 in each type of breast cancers, compared with normal breast cancer tissues, we also found ZNF213 was increased in every subtype form the TCGA database (https://tcga-data.nci.nih.gov/docs/publications/tcga/) (**Figure 1E**). Then we analyzed the prognostic impact of ZNF213 in breast cancer samples (https://kmplot.com). However, ZNF213 expression did not correlate with the progression-free survival in both ER alpha positive and ER alpha negative patients (**Figures 1F–G**). However, ZNF213 expression specifically relates to poor prognosis in endocrine therapy patients (**Figure 1H**).

### ZNF213 Is Required for Cell Proliferation and ER Alpha Signaling in Breast Cancer Cells

**Figure 2:**
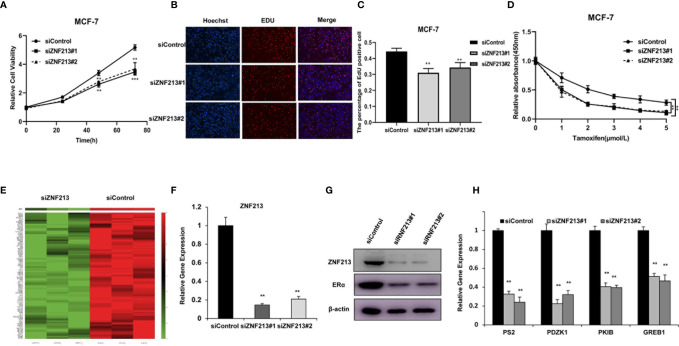
ZNF213 is required for cell proliferation and ER alpha signaling in breast cancer cells **(A)** Depletion of ZNF213 inhibits the proliferation in MCF-7. Cells were transfected with siControl or siZNF213. After 24 h, the assay was CCK8 used to determine the cellar metabolic activity at indicated time points after infection. Experiments were done in triplicates. **P < 0.01; ***P < 0.001 for cell growth comparison. **(B, C)** DNA synthesis assessed using EdU assay in MCF-7 transfected with siControl or siZNF213. Experiments were done in triplicates. **P < 0.01 for cell growth comparison. **(D)** Comparison of tamoxifen sensitivity between siControl and siZNF213. MCF-7 cells were transfected with siZNF213 or siControl. After 24 h, cells were plated into 96-well plate, while each well contained 4,000 cells. The indicated tamoxifen concentrations were used for 48 h. The numbers of the cells were determined *via* CCK8 kit for the cellar metabolic activity. Experiments were done in triplicates. **P < 0.01; for cell growth. **(E)** The heat-map graph shows the ER alpha regulating genes, which is significantly inhibited by ZNF213 depletion in MCF-7 cells. The pathway-enrichment analysis was used by the threshold P < 0.001 and fold change >2 to derive regulated genes. ZNF213 was depleted by siRNA (mix of siZNF213 #1 and siZNF213 #2) or treated with siControl. After 48 h, the whole mRNA was extracted for RNA sequence analysis. The siControl and siZNF213 were done in triplicates. **(F)** ZNF213 depletion effect by two different siRNA oligos. MCF-7cells were transfected with two independent ZNF213 siRNAs or siControl. The relative ZNF213 mRNA levels were determined by QPCR. **(G)** ZNF213 depletion effects on ER alpha protein level by two different siRNA oligos. MCF-7cells were transfected with two independent ZNF213 siRNAs or siControl. ZNF213 and ER alpha protein levels were determined by Western blot analysis. Actin was used as internal control. **(H)** ZNF213 depletion decreases ER alpha target genes. MCF-7 cells were transfected with siZNF213 or siControl. After 48 h, total RNA was prepared and the expression of the endogenous ER alpha target genes, PS2, GREB1, PKIB, and PDZK1 were determined by qPCR. Shown are the results from three experiments. **P < 0.01 for target gene expression comparison.

We further analyzed the role of ZNF213 in cell proliferation. The CCK8 assay showed that ZNF213 depletion significantly inhibited cell proliferation in both MCF-7 (**Figure 2A**) and T47D cells (**Figure S1A**). An EdU incorporation assay also revealed that the proliferation of MCF-7 (**Figures 2B, C**) and T47D cells was impaired with the knockdown of ZNF213 (**Figures S1B–C**). Furthermore, we also found that depletion of ZNF213 significantly decreased cell migration capacity in both MCF-7 and T47D cells (**Figures S2A–D**). To further validate that ZNF213 is relevant for endocrine resistance, MCF-7 and T47D cells were treated with the indicated tamoxifen concentrations, we found that ZNF213 depletion sensitized tamoxifen inhibition effect in MCF-7 and T47D breast cancer cells (**Figure 2D**, **Figure S1D**). To analyze the role of ZNF213 in breast cancer cells in an unbiased way, we depleted ZNF213 in MCF-7 cells for RNA sequencing analysis. The whole genomic profiling indicated that ZNF213 decreased a group of ER alpha target genes expression (**Figure 2E**). This data might indicate ZNF213 modulated ER alpha signaling in breast cancer cells. To prove this hypothesis, we used two independent siRNAs to carry out the experiments. **Figure 2F** showed that the siRNAs show high knockdown efficiency in MCF-7 cells. The immunoblotting indicated that ZNF213 depletion significantly decreased ER alpha protein level (**Figure 2G**). Besides, QPCR assay shows that ZNF213 depletion decreased the expression of ER alpha target genes, including PS2, PDZK1, PKIB, and GREB1 (**Figure 2H**).

### ZNF213 Depletion Inhibits ER Alpha Protein and ER Alpha Target Genes in Breast Cancer Cells

**Figure 3:**
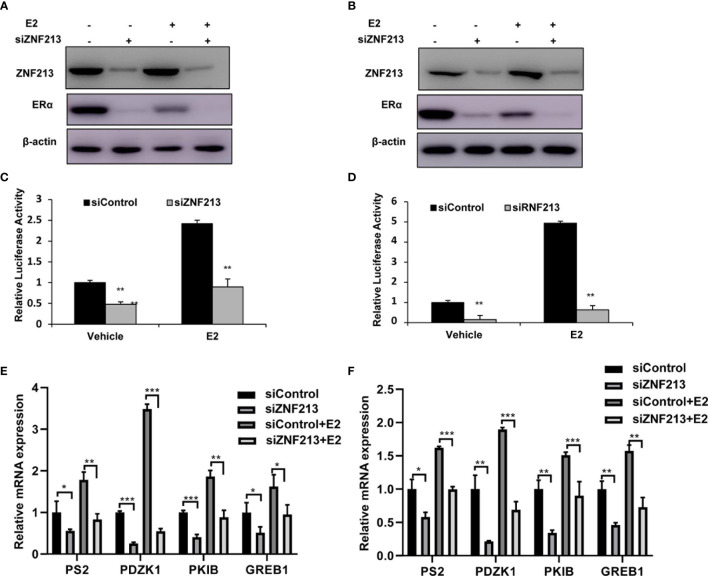
ZNF213 depletion inhibits ER alpha protein and ER alpha target genes in breast cancer cells **(A, B)** ZNF213 depletion effect on ER alpha protein level in MCF-7and T47D cells. Cells were transfected with siZNF213 or siControl. After 48 h, cells were treated with either ethanol or 10 nM estradiol for 6 h. ZNF213 and ER alpha protein levels were determined by Western blot analysis. Actin was used as internal control. **(C, D)** ZNF213 depletion affects ERE-luciferase activity in MCF-7and T47D cells. Cells were transfected with siZNF213 or siControl together with ERE luciferase reporter plasmid. Cells were treated with 10 nM estradiol or vehicle. Luciferase activity was measured 48 h after transfection. Shown are the results from three experiments. **P < 0.01 for luciferase activity comparison. **(E, F)** ZNF213 depletion decreases ER alpha target genes in MCF-7and T47D cells. Cells were transfected with siZNF213 or siControl. After 48 h, cells were treated with either ethanol or 10 nM estradiol for 6 h. Total RNA was prepared and the expression of the endogenous ER alpha target genes, PS2, GREB1, PKIB, and PDZK1 were determined by qPCR. Shown are the results from three experiments. *P < 0.05; **P < 0.01; ***P < 0.001 for target gene expression comparison.

We further tested ZNF213 effect on ER alpha signaling in both vehicle and E2-treated conditions. ZNF213 depletion could decrease ER alpha protein level in vehicle and E2-treated conditions in both MCF-7 and T47D cells (**Figures 3A, B**). In order to determine if ZNF213 knockdown could affect ER alpha transcriptional activity, we measured estrogen response element (ERE) luciferase activity in both MCF-7 and T47D cells. The luciferase assay showed that ZNF213 depletion decreased ERE luciferase activity in both MCF-7 and T47D cells (**Figures 3C, D**). Consistently, ZNF213 depletion could dramatically decrease ER alpha target gene expression in MCF-7 and T47D cells, including PS2, GREB1, PKIB, and PDZK1 (**Figures 3E–F**).

### ZNF213 Could Associate With ER Alpha in Breast Cancer Cells

**Figure 4:**
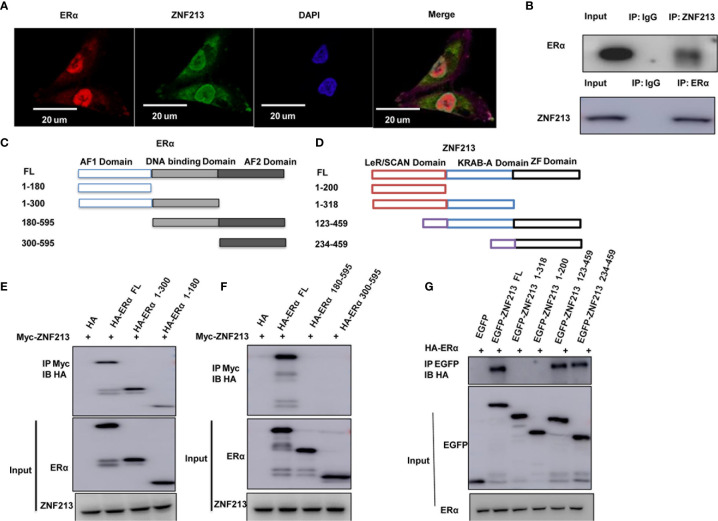
ZNF213 could associate with ER alpha in breast cancer cells **(A)** Intracellular localization analysis of ZNF213 and ER alpha by immunofluorescence assay. MCF7 cells were cultured in normal medium before fixation. Intracellular localization of ER alpha (green) and ZNF213 (red) were shown. Nuclei (blue) were stained with 4’,6-diamidino-2-phenylindole (DAPI). **(B)** Co-IP assay reveals association between endogenous ZNF213 and ER alpha in MCF7 cells. MCF-7 cells were harvested with RIPA lysis buffer. CO-IP was performed using antibody as indicated. **(C)** ER alpha domain structure and deletion mutants used in the study (Full length, ΔAF1, ΔAF1+ΔDBD, ΔAF2, ΔAF2+ΔDBD). **(D)** ZNF213 full length and deletion mutants are used in the study (Full length, ΔZF domain, ΔLeR/SCAN domain, ΔZF+ΔKRAB-A domain). **(E, F)** AF1 domain is required for ER alpha to interact with ZNF213 **(G)** ZF domain is required for ZNF213 to interact with ER alpha.

We further investigated the localization of ZNF213 and ER alpha in breast cancer cells. The immuno-staining showed that ER alpha mainly located in the nuclear, while ZNF213 located both in the cytosol and nuclear (**Figure 4A**). The endogenous immuno-precipitation showed that ZNF213 could interact with ER alpha in MCF-7 cells (**Figure 4B**). ER alpha was composed of three functional domains: AF1 domain, DNA binding domain, and AF2 domain (**Figure 4C**), while ZNF213 was composed by LeR/SCAN domain, KRAB-A domain, and ZF domain (**Figure 4D**). We made the deletion constructs of ER alpha and ZNF213 to further characterize the interaction domain. The immuno-precipitation assay showed that the AF1 domain of ER alpha was required for its interaction with ZNF213, while the ZF domain of ZNF213 was responsible to associate with ER alpha (**Figures 4E–G**).

### ZNF213 Modulates ER Alpha Poly-Ubiquitination and Protein Stability

**Figure 5:**
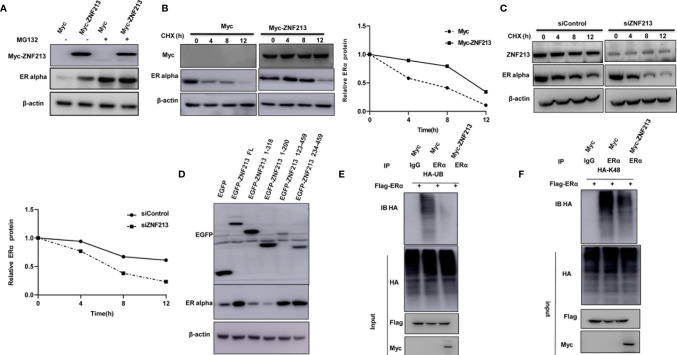
ZNF213 modulates ER alpha poly-ubiquitination and protein stability **(A)** In the presence of the proteasome inhibitor MG132, the stabilization effect of ZNF213 on ER alpha did not further increase ER alpha protein levels. HEK293 cells were transfected with 2 µg ER alpha plasmid and 0.5 µg Myc-tag or Myc-ZNF213 plasmids. After 24 h, cells were treated with 10 uM MG132/vehicle for 6 h. Cell lysates were prepared for Western blot analysis. The results are representative for three independent experiments. **(B)** ZNF213 increases ER alpha half-life in HEK293 cells. HEK293 cells were transfected with HA-ER alpha plasmid and Myc-tag or Myc-ZNF213 plasmids. After 24 h, cells were treated with 100 µM cycloheximide/vehicle for indicated times. Cell lysates were prepared for Western blot analysis. The results are representative for three independent experiments. The ER alpha relative density was measured by Image J software. **(C)** ZNF213 increases ER alpha half-life in MCF-7 cells. Cells were transfected with siZNF213 or siControl. After 48 h, cells were treated with 100 µM cycloheximide/vehicle for indicated times. Cell lysates were prepared for Western blot analysis. The results are representative for three independent experiments. The ER alpha relative density was measured by Image J software. **(D)** The ZF domain is required for ZNF213 to stabilize ER alpha in HEK293 cells. **(E)** ZNF213 prohibits ER alpha poly-ubiquitination. HEK293 cells were transfected with 2 µg ER alpha plasmid and 0.5 µg Myc-tag or Myc-ZNF213 plasmids. After 24 h, cells were treated with 10 uM MG132 for 6 h. Cells were directly harvested and Western blot analysis using ER alpha antibody was used to detect ubiquitinated ER alpha forms. The predicted molecular weight of poly-ubiquitinated ER alpha is indicated. **(F)** ZNF213 decreases K48-linked poly-ubiquitination of ER alpha. HEK293 cells were transfected with 2 µg ER alpha plasmid, 0.5 µg HA-K48 Ubi plasmid, and 0.5 µg Myc-tag or Myc-ZNF213 plasmids. The cell extracts were immuno-precipitated with HA antibody. The K48 specific poly-ubiquitinated ER alpha was detected *via* western blotting analysis.

Since ZNF213 could associate with ER alpha in breast cancer cells, we further investigated the biological effect of such interaction. Co-transfection of ER alpha and ZNF213 in HEK293 cells showed that ZNF213 could increase ER alpha protein level, which effect could be minimized with the presence of the proteasome inhibitor MG132 (**Figure 5A**). The protein half-life assay showed that ZNF213 could increase the protein stability of ER alpha (**Figures 5B, C**), while co-transfection with ZNF213 deletion variants showed that the ZF domain was required for the stabilization effect for ER alpha protein (**Figure 5D**). We further investigated the effect of ZNF213 on ER alpha ubiquitination. The ubiquitination assay showed that ZNF213 could significantly inhibit ER alpha overall ubiquitination level and K48-linked ubiquitination (**Figures 5E, F**).

## Discussion

**Figure 6:**
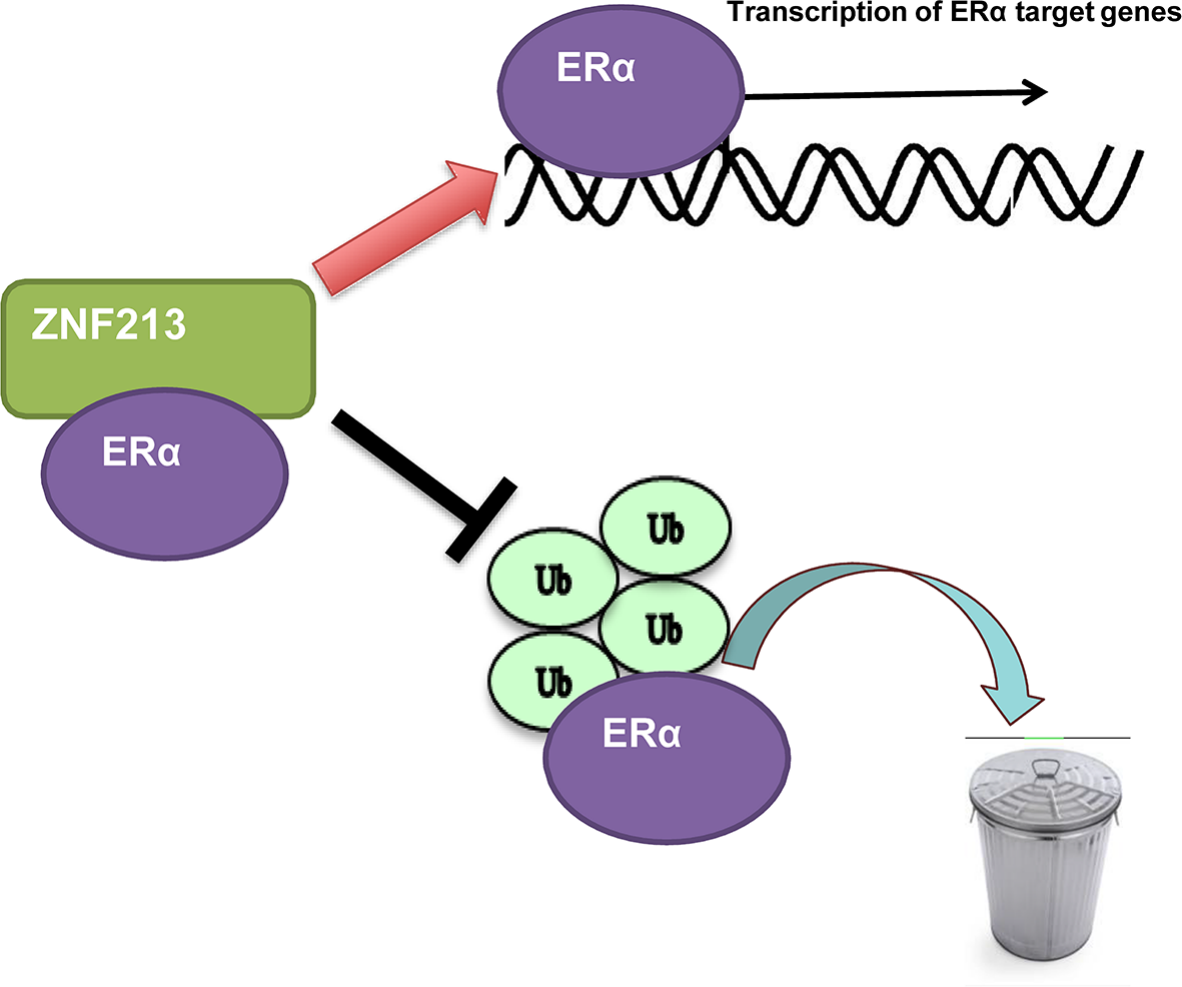
The hypothetic model of ZNF213 in modulating ER alpha signaling in breast cancer: ZNF213 associates with ER alpha and facilitates ER alpha stability *via* inhibiting ER alpha poly-ubiquitination and degradation.

In this study, we identified one Zinc finger protein ZNF213, which was higher expression in human breast cancer samples, promoted ER alpha signaling activity and ER alpha stability in breast cancer cells. ZNF213 associated with ER alpha and inhibited ER alpha poly-ubiquitination and degradation (**Figure 6**).

The importance of ER alpha signaling has been identified for 30 years (26), based on the fact that ER alpha is one of the most important drivers for breast cancer progression. Although ER alpha mutation is not common, the elevated expression of ER alpha could be found in most of human breast cancers (10). Based on current knowledge, ER alpha signaling is a suitable target for breast cancer therapy. Selective ER alpha modulators, such as tamoxifen, are effective in blocking ER alpha signaling and breast cancer progression. However, most of patients will development endocrine resistance, making it a critical clinical issue for breast cancer therapy. Based on the published literatures, most of resistant breast tumors still maintain ER alpha expression (27). There are several possible mechanisms for endocrine resistance. For example, ER alpha could trans-activate other growth factor pathways, such as IGF and EGFR signaling, to overcome endocrine resistance (28). Besides, several modifications, including ubiquitination, acetylation, and phosphorylation, could enhance ER alpha signaling strength and overcome tamoxifen resistance (15, 21, 29). Based on these mechanisms, modulating ER alpha protein stability could a plausible strategy for therapeutics in ER alpha positive breast cancer patients.

Recently studies have identified several RING finger proteins or Zinc finger proteins in modulating ER alpha stability and breast cancer progression. For example, RNF31 could associate with ER alpha and promote ER alpha stability *via* inducing ER alpha mono-ubiquitination (30). Besides, RNF8 was also found to co-activate ER alpha target genes and prolong ER alpha half-life in breast cancer cells (14). When it comes to ZNF213, which belongs to zinc finger protein members, we firstly identified its role in breast cancer. ZNF213 was firstly regarded as a modulator for gene expression, based on its capability to associate with DNA (31). However, our study showed that ZNF213 could associate with ER alpha protein and promotes ER alpha function. The co-transfection in HEK293 cells indicated the modulation effect of ZNF213 on ER alpha signaling went through protein stability, not through direct genomic regulation. These findings provided a novel insight of ZNF family members in modulating estrogen signaling and breast cancer progression. ZNF213 could be a novel target for ER alpha positive breast cancer patients.

## Conclusions

In summary, our study provided a novel regulatory mechanism between ZNF213 and ER alpha in breast cancer cells. The zinc finger protein ZNF213 modulated ER alpha signaling and breast cancer progression through a post-translational mechanism. Our study implicated the important role of ZNF213 in ER alpha signaling and improved the understanding of ZNF213 in both genomic and non-genomic regulation in human cancer. As such an important regulator of ER alpha signaling, ZNF213 could be a promising target for ER alpha positive breast cancer therapeutics.

## Data Availability Statement

The datasets presented in this study can be found in online repositories. The names of the repository/repositories and accession number(s) can be found in the article/**Supplementary Material**.

## Author Contributions

HY and XLL performed most of the benchwork. XL and ZN participated in western blot, real time PCR work. TW provided reagents and advice. LM, TZ, and QH supervised the process of the study and performed the manuscript writing. All authors contributed to the article and approved the submitted version.

## Funding

Tianjin Research Innovation Project for Postgraduate Students, HY. The project was supported from 111 Project (Grant No. D20036). The National Natural Science Foundation of China (Grant No. 81702725), The Program for Science & Technology Innovation Talents in Universities of Henan Province (Grant No. 21HASTIT049), TZ. The National Natural Science Foundation of China (Grant No. U1804167, Grant No. 81770721. Grant No. 81570624), QH. Key Scientific and Technological Projects of Henan Province (Grant No. 202102310024), LM. Key Scientific Research Projects of Higher Education Institutions in Henan Province (Grant No.18A320004), LM.

## Conflict of Interest

The authors declare that the research was conducted in the absence of any commercial or financial relationships that could be construed as a potential conflict of interest.

## Supplementary Material

Supplementary Figure 1 ZNF213 is required for cell proliferation in breast cancer cells. **(A)** Depletion of ZNF213 inhibits the proliferation in T47D. Cells were transfected with siControl or siZNF213. After 24 h, the assay was CCK8 used to determine the cellar metabolic activity at indicated time points after infection. Experiments were done in triplicates. **P < 0.01; ***P < 0.001 for cell growth comparison. **(B, C)** DNA synthesis assessed using EdU assay in T47D. Cells were transfected with siControl or siZNF213. Experiments were done in triplicates. **P < 0.01 for cell growth comparison. **(D)** Comparison of tamoxifen sensitivity between siControl and siZNF213. T47D cells were transfected with siZNF213 or siControl. After 24 h, cells were plated into 96-well plate, while each well contained 4,000 cells. The indicated tamoxifen concentrations were used for 48 h. The numbers of the cells were determined *via* CCK8. Experiments were done in triplicates. **P < 0.01 for cell growth.

Supplementary Figure 2 ZNF213 is required for cell migration in breast cancer cells. **(A–D)** Wound healing assay of MCF-7and T47D cells transfected with the indicated siRNA. Quantification of wound closure at the indicated time points. Data are presented as ± SD. **P < 0.01 (student’s t-test).

## Abbreviations

ZNF213, zinc finger protein 213; AF1, transcriptional activation domain 1; DBD, DNA binding domain; AF2, transcriptional activation domain 2; ER alpha, estrogen receptor alpha; HER2, human epidermal growth factor receptor 2; PR, progesterone receptor; TNBC, triple negative breast cancer; RBR, RING-between-RING domain; ZF, zinc finger domain; EMT, epithelial-mesenchymal transition; ATCC, American Type Culture Collection; QPCR, Real-Time Quantitative PCR.
